# A first case report of hypohidrotic ectodermal dysplasia from Oman

**DOI:** 10.1002/ccr3.2723

**Published:** 2020-02-29

**Authors:** Musallam Al‐Araimi, Nishath Hamza, Aliya Al Hosni, Hiba Al Mazrooey

**Affiliations:** ^1^ National Genetic Centre Royal Hospital Muscat Oman

**Keywords:** ectodermal dysplasia, genetic, mutation

## Abstract

This is a first case report of a patient with hypohidrotic ectodermal dysplasia from Oman, who was found to carry a mutation in the EDAR gene after candidate gene selection based on regions of homozygosity in his genome.

## Graphical abstract

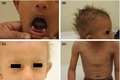

## 1 CASE REPORT

Hypohidrotic ectodermal dysplasias (HED) are a group of developmental genetic disorders which primarily affect tissues of ectodermal origin such as teeth, skin, hair, nails, mucous membrane, and sweat glands. HEDs are mostly inherited as X‐linked recessive disorders (OMIM: 305 100) as the result of mutations in the ectodysplasin A (EDA) gene.^1^ However, HDE can also be inherited in autosomal dominant (OMIM: 129 490) and autosomal recessive manners (OMIM: 224 900) due to mutations in the ectodysplasin A receptor (EDAR) and EDAR‐associated death domain (EDARADD) genes. These three genes encode proteins which activate the nuclear factor‐κB (NF‐κB) signaling pathway, which is critical for normal development of ectodermal organs both in humans and in mice.^2^ Two other genes, WNT10A and TRAF6, have also been associated with HED.^3^ A fourth rare HED subtype where the most significant clinical presentation is immunodeficiency (HED‐ID) was also reported to be associated with hypomorphic mutations in the coding region of the IKBKG (or NEMO) gene (Xq28) or rarely, mutations in the NFKBIA gene (14q13), both of which are involved in NF‐κB activation.^4^, ^5^

**Figure 1:**
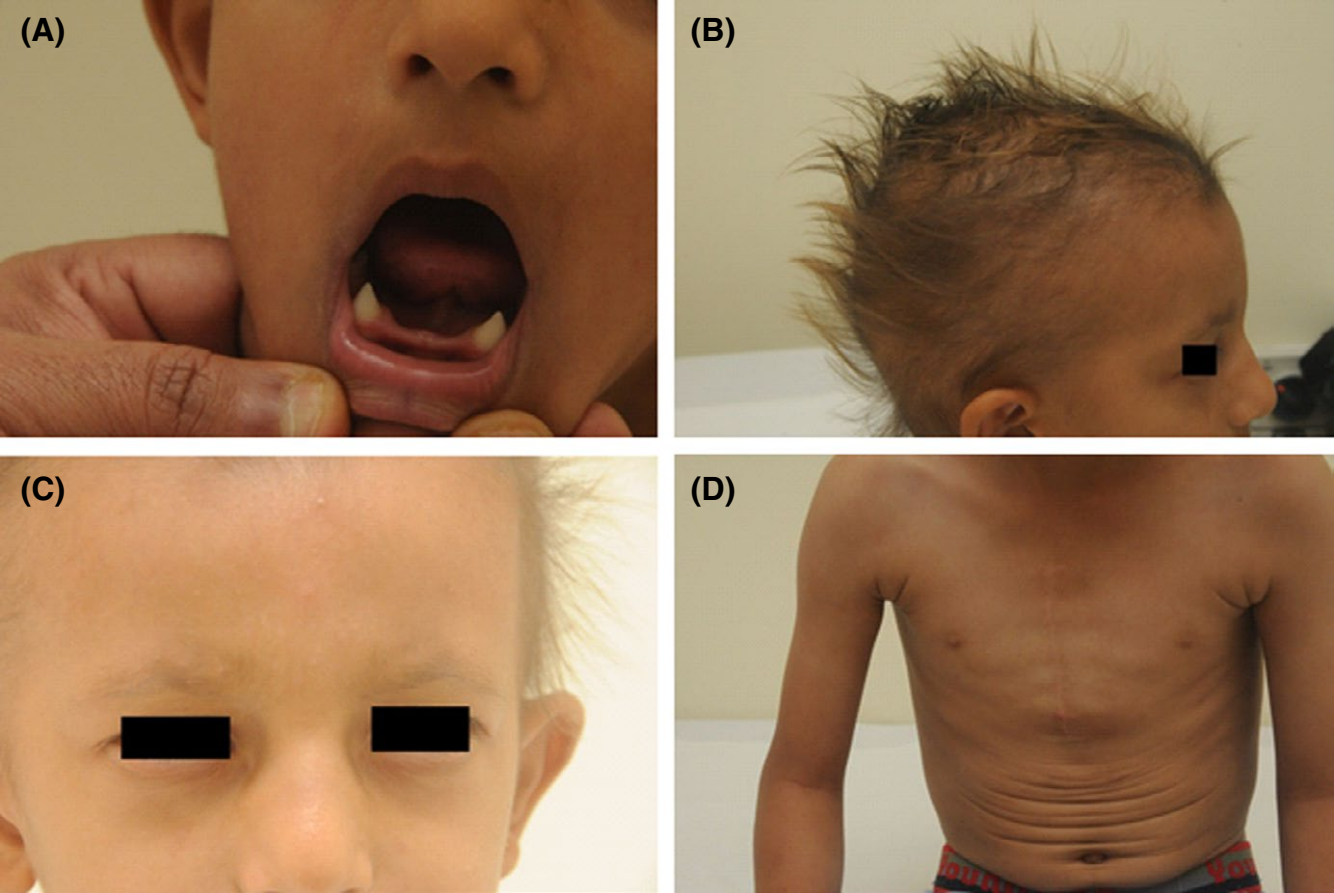
Clinical presentation of HED patient. A, Pointed incisors; B, Sparse light‐colored scalp hair; C, Absent eyebrows and eyelashes, periorbital wrinkles, saddle‐shaped nose; D, Wrinkled and thin skin

We present here the first case report of an HED patient from Oman. Our index patient is a five‐year‐old male who was referred to our genetic clinic at two years of age with large secundum atrial septal defect, abnormal hair and alopecia, sparse eyebrows, thin and wrinkled skin, bluish sclera, saddle‐shaped nose, pointed upper teeth (hypodontia), severe intolerance to heat, impaired ability to sweat, chronic paronychia, developmental delay, and failure to thrive (Figure 1). The index patient was quite thinly built and had failed to walk independently until 21 months of age. When he was 2 years of age, our patient underwent a successful surgical procedure for atrial septal defect closure. At 3 years of age, he had a history of unsteady gait and infrequent falling. He also seemed to possess reduced immunity as was apparent from delayed wound healing and was also MRSA (methicillin‐resistant Staphylococcus aureus) positive. The index patient is the product of a consanguineous union and has a normal, healthy 8‐year‐old female sibling. The parents denied any family history of developmental disorders. The DNA of the index patient was extracted from his blood.

Although the patient was suspected to have a form of HED, he exhibited clinically more severe syndromic presentations than HED patients reported previously. This led us to consider the possibility of microdeletions that may affect relevant genes. To exclude this possibility, the patient's DNA sample was first analyzed using comparative genomic hybridization (CGH) array on the Affymetrix platform using the CytoScan HD kit at our center. Data analysis carried out using the CHAS software (v.3.1.0.15) did not indicate any copy number variants. However, probing for regions of homozygosity (ROH) based on genome‐wide single nucleotide polymorphisms (SNPs) uncovered multiple stretches of homozygosity. On analyzing the genes within these ROH regions using the Human Phenotype Ontology tool, we honed in on the EDAR gene at the 2q13 locus as a candidate for further analysis. The index patient DNA was then subjected to full gene sequencing of the EDAR gene, which is associated with autosomal recessive ectodermal dysplasia, type 10B, and was found to carry the homozygous variant c.73C > T, p.Arg25Ter (NM_022336.3) in the EDAR gene. The EDAR:c.73C > T variant was reported previously in a 6‐year‐old female patient, albeit in heterozygous form and associated with autosomal dominant HED, type 10A.^6^

The c.73C > T, p.Arg25Ter in the EDAR gene, creates a stop codon within exon 3 of the coding region. This is predicted to cause premature truncation of the EDAR protein and result in the complete deletion of the transmembrane and binding domains which are critical to the EDAR protein's function as a soluble ligand ectodysplasin A receptor and its signaling activity, respectively. The complete loss of protein function due to the c.73C > T mutation and the consequent effect on gene dosage is a likely explanation for why our patient with the homozygous form of this mutation exhibits a more severe clinical presentation than the previously reported patient who presented with tooth agenesis as its major clinical presentation attributed to the heterozygous form of the c.73C > T mutation.^6^

**Figure 2:**
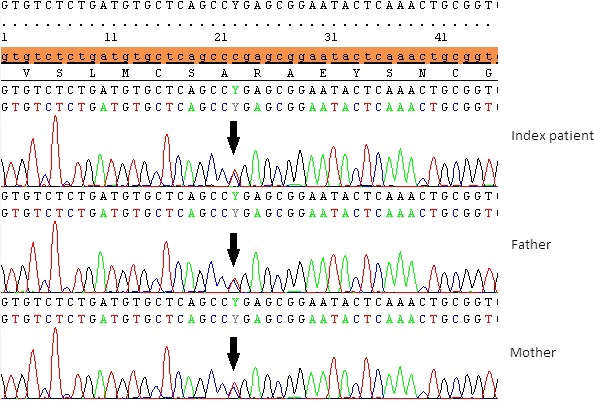
Sanger sequencing analysis of the EDAR gene. A sequence chromatogram showing the mutation profile in the index patient and his parents

Screening for the c.73C > T mutation in the parents and normal sibling of the index patient using exon 3‐specific Sanger sequencing indicated that all of them were heterozygous carriers of this mutation (Figure 2). These observations further implicated the pathogenic role of the EDAR gene: c.73C > T variant in the HED phenotype of our index patient. We then provided effective genetic counseling sessions to the family, whereby intervention through pre‐implementation genetic diagnosis was offered to the family as an option and all their queries were responded to adequately.

The current management of the index patient is focused on providing parental guidelines for better adaptation to climate changes to overcome the problems from hypohidrosis (inability to sweat), the use of moisturizing ointments regularly, routine hydration, and avoidance of intense physical exertion to avoid excessive sweating. The patient was also recruited into a comprehensive care and management follow‐up schedule with specialist clinics in dermatology and dentistry.

## CONFLICT OF INTEREST

All authors attest that they have no conflict of interests to declare.

## AUTHOR CONTRIBUTIONS

MAA: conducted clinical sampling, patient counseling and manuscript review. MH: carried out data analyses and wrote the manuscript. AAH: carried out molecular genetic analyses. HAM: carried out molecular cytogenetic analyses.
